# Odontological Society of Pennsylvania

**Published:** 1890-05

**Authors:** 


### ODONTOLOGICAL SOCIETY OF PENNSYLVANIA.

THE regular meeting of the Odontological Society of Pennsylvania was held Saturday evening, January 4, 1890, at the hall, Thirteenth and Arch Streets. President Truman in the chair. A paper was read on "The Dental Crown,"[1] by W. H. Gates, D.D.S., Philadelphia (see page 257); also one by C. H. Littleton, D.D.S., Philadelphia, on "A New Reamer for Pulp-Canals," as follows:

There is no operation in dentistry which requires more skill and care than the treatment of the roots of devitalized teeth, and yet, judging from the various methods advocated, the dental profession is far from a satisfactory solution of the problem.

It is not the intention of this paper to discuss the different conditions requiring root treatment, but only to present some new facilities for the preparation of the canal and hermetical sealing of the apical foramen.

---

[1] The discussion of this paper was deferred until Dr. Littleton's paper had been read, and both papers were then discussed together.

To insure permanent success, it is necessary, first, to carefully remove the entire contents of the canal without forcing any substance whatever through the foramen; secondly, the canal must be sufficiently enlarged at the apex to enable the operator to make a filling at that point with a strictly impervious, non-absorbing material.

To accomplish this an instrument is necessary that will cut smoothly, clearing itself as it advances, without any piston-like effect. The instruments I use for this purpose are self-centring in the canal without the usual guide-point. The ordinary point on a canal reamer has a tendency to force material in advance instead of withdrawing it, and it also retards the cutting. The only bearing of this reamer is its rounded front, which, by being short, enables it to centre itself in the canal without interfering with the flexibility of the stem. This front is cut into very definite blades for rapid cutting and self-clearing; and the general form of the reamer, being a flattened globe, serves to withdraw the cuttings.

The longest diameter of a pulp-canal reamer should be at right angles to the stem, in order to permit the flexible stem to control it from its front instead of from the rear. Thus it is always free from the cramped position of a long bodied reamer and the consequent danger of breakage.

The stem should have a fine spring temper, so as to operate easily at any required angle,—its flexibility varying according to the directness of the canal. For the more crooked canals it should have a uniform flexibility extending two inches from the cutting end.

The handle is octagon in shape, which, in connection with the flexible stem and cutting front of the reamer, makes this hand instrument so delicately sensitive to the touch of the operator that he is enabled to feel its advance in the canal, and, by the greater resistance, judge of its approach to the end of the root. I have them made in sizes varying from 1 to 4.

To hold the flexible stem at the necessary curve, and thus relieve the reamer from side-pressure when operating in the roots of posterior teeth, I have a hole made through the centre of a mouth-mirror; the stem passing through from the back can thus be controlled by the handle of the mirror.

For convenience in placing the flexible stem in this position a narrow slot is cut in the mirror from the side opposite the handle. Hooks soldered to the rim of the mirror will also further assist in guiding the instrument.

In reaming, or in any operation in a pulp-canal, it is of the first importance never to produce a pneumatic or piston-like effect. Periodontitis is often produced from this cause alone, as a bubble of air, charged as it is apt to be with septic matter, is highly irritating, and its imprisoned elastic force, if maintained, is more hurtful to the peridental membrane than a puncture by an instrument. It is, therefore, obvious that thorough desiccation of the canal is an essential prerequisite. With perfect dryness and the self-clearing action of this instrument no material whatever will be forced through the foramen.

In operating in a small and crooked canal, as, for instance, in an anterior lower molar, the approach at the mouth of the canal should be straightened with the engine-bur, cutting in that case very freely towards the front walls.

To ream a canal with safety and facility the reamer must be proportioned to the size of the canal. Having followed a very fine canal with reamer No. 1 a short distance,—thus obtaining greater freedom for the flexible stem,—it is easily enlarged with No. 2.

When the end of the root has been reached with this reamer, there is a definite shoulder formed at that point, against which an amalgam filling not larger than a small pin-head can be easily made. The amalgam should have such plasticity that a small portion may be carried on one of these reamers and tamped gently to place. Amalgam is well adapted for this purpose because so little force is needed to make it an hermetical stopping, and, being non-porous, it cannot become charged with septic matter. As cotton, the cements, and especially gutta-percha absorb the matter that remains more or less present in all cases, the necessity of impervious sealing at the apex is clearly indicated. The apical foramen being sealed with amalgam, the remainder of the canal can be filled with cotton covered with gutta-percha, or other material easy of removal, if future operations in the canal be found necessary. Should it ever be necessary to reopen the foramen, the small amalgam filling can be readily cut away with one of these reamers.

In case of an enlarged foramen, from whatever cause, the softened dentine and cementum can be removed with one of these instruments, and a shoulder formed near the end of the root with the next in size, against which a plug of platinized silver wire covered with amalgam may be adjusted.

The amalgam filling, made at the end of the root, affords great advantage when the root is used for crown- and bridge-work, enabling the deeper anchorage of the pin and preventing any disturbance of the peridental membrane during insertion or removal.

DISCUSSION.

*Dr. J. A. Woodward.*—The reaming of root-canals has been as strongly advocated as objected to by many competent operators. The practice of those who do not ream is largely the result of the use of defective instruments. After a struggle to remove the tempered point of a drill from a canal difficult of approach, most of us are careful not to have that experience repeated, and, to avoid it, only ream or enlarge a canal as far as we can perfectly control the instrument used.

The set of reamers presented to us to-night is a decided advance. The slender, flexible shank permits the cutting blades to follow a canal, when the reamer is of proper size in relation to the diameter of the canal, and should give us the means of safely reaming where we would not venture with the older drills. The perforated mirror is an ingenious assistant. As to reaming to the apex of all roots, I have been led by experience not to try it. Generally I ream sufficiently to gain access near to the apex. I have found that the less the canal at the apex is interfered with the better has been my success.

My experience with crown- and bridge-work has been quite limited, but if for an abutment for a bridge or not I think all roots, with few exceptions, should be so treated that the canal-filling should not need to be removed, as removable root-fillings appear to invite the very conditions we wish most to avoid. The use of gutta-percha to make the joint between the new crown of Dr. Gates and the root I can unreservedly endorse, as it has proved with me the best material for that purpose.

*Dr. Register.*—I do not think it matters much whether you ream or not. In reference to root-fillings we are not able yet to distinguish just which roots should be filled with cotton, which with gutta-percha, and which with amalgam at the apex. If the pulp has been removed, I prefer to fill the tooth with amalgam even to the apex. I am much pleased with the instrument and think it a very good idea.

*Dr. Roberts.*—If I understand it right, the doctor claims that by the flexibility of the stem of his instrument he gets a delicacy of touch far greater than with the ordinary instrument, and which enables him to follow any canal and prevents any mistake.

*Dr. Littleton.*—Dr. Roberts grasps the idea. If there is any canal, that instrument will follow it, and follow it to the apex. The instrument is very delicate to the touch. Then, the great advantage is that it does not push matter ahead of it and carries no septic matter into the canal. If we have to reach the apex of a bad root in a tortuous canal, while this reamer will follow the canal it will not push matter in advance of it.

*Dr. Jamison.*—When I first began to practise dentistry I used to feel along with a flexible instrument which would cut the canal open; but now I have no use for it. From the instrument exhibited it looks very much to me that it cannot but force matter ahead of it from the twist which it has.

*Dr. Littleton.*—If the doctor will take my instrument and try it awhile I think he will change his mind and will find it quite different from what he anticipates.

*Dr. Faught.*—Some six weeks ago I had the pleasure of giving some statistics to a society in New York on "whether to ream or not to ream," and I can assure you it is a moot question, and not at all decided that we should always ream. I would like to know the practice of each, and hope all present will state in their discussion whether they are in the habit of reaming. I let the apex alone after I have cleaned out the root-canal. It may be some advantage to the teeth to fill the apex with cotton, gutta-percha, or what not, but I fail to see it. When I wish to ream a canal I can feel with a flexible instrument as well as I can with a flexible point.

*Dr. Tees.*—I am pleased with Dr. Littleton's reamer and think it is a good instrument. The fault of Dr. Gates's crown is that the post comes to the labial and not the palatal surface of the tooth. I think an offset of $\frac{1}{32}$ to $\frac{1}{16}$ of an inch on the next tooth will serve very little to hold it in place.

*Dr. Gates.*—The anatomy of the tooth will determine the direction of the post.

*Dr. Tees.*—This crown is some advance upon posts for the teeth.

*Dr. Bonwill.*—It is so seldom that we hear of a new thing with any real merit in it that we are always inclined to doubt the usefulness of it. But I am inclined to think well of Dr. Gates's crown. I have crowned teeth which were used for five years, and no trouble was experienced with them.

*Dr. Truman.*—In regard to Dr. Gates's crown it is evidently an advantage to have the post or pivot on the concave side of the root.

When properly applied with the instrument which Dr. Gates has described it is undoubtedly a great improvement. I regard it as a useful addition to prosthetic dentistry.

INCIDENTS OF OFFICE PRACTICE.

*Dr. Head.*—I have been noticing for a year or two past that something has been making fearful ravages in the teeth of my patients. I found that on every occasion of this my patient was an ardent lover of "acid phosphate." I felt there must be some connection between this drug and the trouble with the teeth, so I determined to investigate the matter. To do this I got a supply from a druggist and dropped a tooth into it, and found that in thirty minutes it had formed a thin film on the outside of the tooth and the whole enamel was softened. In two days the enamel was as soft as the other part of the tooth, and the whole tooth could be crumbled away.

Such being the case, I thought it best to report it to this society, that others having patients using this drink might warn them of its deleterious effects.

*Dr. Truman.*—I have heard the same complaints made against this drug, and feel that we all should warn our patients against its use.

